# Inhibition of Aurora B by CCT137690 sensitizes colorectal cells to radiotherapy

**DOI:** 10.1186/1756-9966-33-13

**Published:** 2014-01-29

**Authors:** Xiaoyu Wu, Wentao Liu, Qinhong Cao, Che Chen, Zhiwei Chen, Zhe Xu, Weisu Li, Fukun Liu, Xuequan Yao

**Affiliations:** 1 Department of Surgical Oncology, Affiliated Hospital of Nanjing University of Traditional Chinese Medicine, 155 Guangzhou Road, Nanjing 210029, China; 2 Department of Surgery, Ruijin Hospital, Shanghai Jiaotong University, Shanghai Institute of Digestive Surgery, Shanghai 200025, China

**Keywords:** CCT137690, Aurora B, Radiotherapy, Colorectal cancer

## Abstract

Colorectal cancer is the third most commonly diagnosed cancer worldwide. Although surgery remains the best treatment for this disease, adjuvant chemotherapy and radiotherapy are also very important in clinical practice. However, the notorious refractory lack of responses to radiochemotherapy greatly limits the application of radiochemotherapy in the context of colorectal cancer.

There is a growing interest in the role that Aurora B may play in colorectal cancer cell survival as well as other cancer subtypes. In the current study, we sought to ascertain whether blocking of Aurora B signaling machinery by a small molecule inhibitor, CCT137690, could synergize radiation-induced colorectal cancer cell death. Results showed that CCT137690 increases the sensitivity of SW620 cells to radiation. Mechanistic studies revealed that Aurora B-Survivin pathway may be involved in this synergistic effect.

Taken together, our results for the first time show that Aurora B inhibition and radiation exert a synergistic effect, resulting in enhanced colorectal cancer cell death. This synergistic effect is clinically relevant as lower doses of radiation could be used for cancer treatment, and could provide significant clinical benefits in terms of colorectal cancer management, while reducing unwanted side-effects.

## Introduction

Colorectal cancer (CRC) is the third most commonly diagnosed cancer worldwide with over 1.4 million new cancer cases and 0.6 million estimated deaths every year [1-3]. The incidence of CRC has rapidly increased in China and other Asian countries over the last few decades [4-8], and identifying better ways of treating this cancer is paramount. Although surgery of CRC remains the best treatment, adjuvant chemotherapy and radiotherapy are also very important and beneficial treatments for patients [9,10]. After surgery, chemotherapy and radiotherapy is used to target small cancerous tissues that may be missed during surgery and help to prevent cancer recurrence.

Aurora kinases (of which there are 3 isoforms: Aurora A, B and C) are the most important serine/threonine-protein kinases which regulate the function of centrosomes, spindles, and kinetochores for proper mitotic progression [9,11]. Aurora A overexpression has been observed in various cancers including colorectal cancers. Baba *et. al.* reported overexpression of Aurora A protein in 19% of CRC by immunohistochemistry [12]. High copy amplification of the Aurora A gene was found in colorectal tumors [13] and associated with chromosomal instability phenotypes [14]. In another report, up-regulation of Aurora kinases were detected in 48.5% (97/200) of patients with colorectal carcinoma [15]. Similarly, a previous study reported that the presence of nuclear Aurora B was strongly associated with lymph node metastasis in colorectal cancer [16]. In metastatic colorectal cancer, patients with a high expression level of Aurora B lived significantly shorter compared with patients with a low expression level [17]. Taken together, these studies highlight the association of altered aurora kinases and CRC.

As far as therapeutic options, 5-Fluorouracil (5-FU) remains the most commonly used chemotherapeutic agent for CRC. However, CRC tumors are highly refractory to chemotherapy and many patients eventually relapse. Because of the established roles of Aurora kinases in tumor initiation and progression, many inhibitors of Aurora kinases have been specifically tested for the treatment of colorectal cancers in combination with 5-FU, with some currently in clinical trials [18-22].

Recent studies showed that overexpression of Aurora kinases might have a role in chemo- and radiotherapy resistance of cancers [23,24]. Consistent with this notion, inhibition of Aurora kinases can enhance radiation sensitivity of cancer cells [25,26]. For example, inhibition of Aurora B sensitizes mesothelioma cells by enhancing mitotic arrests [27] and also potently suppresses repopulation during fractionated irradiation of human lung cancer cell lines [28].

CCT137690 is a newly synthesized compound which has been shown to inhibit the activities of Aurora kinases. IC50 values of CCT137690 are 15 and 25 nM for Aurora A and B, respectively. Although CCT137690 has shown promising therapeutic effects on different cancer cells (especially for colorectal cancer) [29-31], a narrow safety margin (due to its activity against hERG ion-channel) may limit its preclinical development [28].

The main cause of treatment failure and recurrence is resistance of cancer cells to radiation and drugs [32,33]. Since inhibition of Aurora kinases can sensitize cancer cells to radiotherapy, it is expected that combining radiotherapy and Aurora inhibition for colorectal cancers may achieve a synergistic therapeutic effects. Concomitant inhibition of Aurora kinases and radiotherapy can also potentially decrease the dosages of either medicine or radiation, which in turns can reduce the side effects of the treatments. Therefore, in our current study we sought to explore whether the combination of radiotherapy with CCT137690 may prove efficacious in the treatment of colorectal cancer cell lines. In this way, optimized combinatorial treatment may lead to a decrease in the requirement of CCT137690 for therapeutic benefit.

## Materials and methods

The authors declared that the current research has been approved by The Ethics Committee of Nanjing University of Traditional Chinese Medicine.

### Reagents

DMEM and fetal bovine serum (FBS) were purchased from Thermo Fisher Scientific at CHINA (Shanghai, China). 3-(4,5-dimethylthiazol-2yl)-2,5-diphenyl tetrazoliumbro-mide (MTT) was obtained from Sigma-Aldrich (Shanghai, China). Anti-Aurora B antibody and anti-Histone H3 (phospho S10) antibody were obtained from Abcam. Anti-Survivin antibody was purchased from Cell Signaling. Anti-Histone H3 and GAPDH antibody were obtained from Santa Cruz Biotechnology.

### Cell culture

The human colorectal adenocarcinoma cell lines, SW48 and SW620, were obtained from the American Type Culture Collection. The cells were maintained in DMEM supplemented with 10% heat inactivated FBS at 37°C, 5% CO_2_, and 95% humidity.

### Plasmids and transfection

The full-length cDNA sequence of survivin was amplified from total RNA of SW620 cells by using Reverse Transcription PCR. The fragment was inserted into pBABE-Puro vector. The control vector plasmid or the plasmid encoding survivin was transfected into Phoenix Retroviral Expression System. Virus was produced and applied onto target cells according to the standard protocol. The cells were subjected to drug-selection for 3 days (0.5 μg/ml of puromycin) to enrich for the desired cells.

### Silencing of Aurora A and B in cells

1.5 × 10^5^ cells were seeded in 60-mm plates and incubated for 24 h before transfection. The negative control siRNA or Aurora A or B siRNA was diluted in Opti-MEM I Reduced Serum Medium and mixed with Lipofectamine 2000 according to the manufacturer’s instructions. The mix of DNA and Lipofectamine was added to cells. After 72 hours post-transfection, expression levels of Aurora genes were determined by Real-time PCR and cells were used for different assays.

### Ionization radiation

Cells were plated in dishes, and then irradiated with X-ray (104.93 cGy/min at 210 kV and 12 mA) by using an X-ray irradiator (MBR-1505R2; Hitachi Medico, Tokyo, Japan) for indicated dosages.

### Determination of surviving fraction

2 × 10^5^ cells were plated in a 60-mm dish. 24 hours later, the cells were exposed to different dosages of ionization radiation. After a 6-hour recovery, one percent of the cells were re-plated in a new dish. After 10 days the number of colonies formed were counted.

### Combination effect of radiation and CCT137690

Cells were first treated with CCT137690 at different concentrations for 48 hours before they were exposed to different dosages of ionization radiation.

### Cell cycle assay

Cells were collected by trypsinization and washed with PBS, centifuged and then resuspended in 0.4 ml of PBS and fixed by adding 1ml cold ethanol slowly. Cells were kept at 4°C overnight. For analysis, cell suspensions were centrifuged at 1500 rpm for 5 mins, washed with PBS and re-suspended in 500 μl staining solution (PBS with 50 μg/ml PI together with 50 μg/ml RNase A) at 37°C for 30 mins in the dark. Cells were analyzed by flow cytometry.

### MTT assay for cell viability

10^4^ cells were seeded into 96-well plates and were treated to either vehicle (DMSO) or different concentrations of CCT137690 for 48 hours. Cell viability was determined and quantified by using MTT assay.

### Guava Nexin assay

The Guava Nexin assay was performed following manufactory protocol (Millipore). Briefly, attached and suspended cells were all collected. Cells were resuspended in 100 μL of medium and incubated together with 100 μL of Guava Nexin Reagent for 20 minutes at room temperature in the dark. Samples then were measured on a Guava System (Millipore). The data were analyzed by using the software provided by the company.

## Results

**Figure 1:**
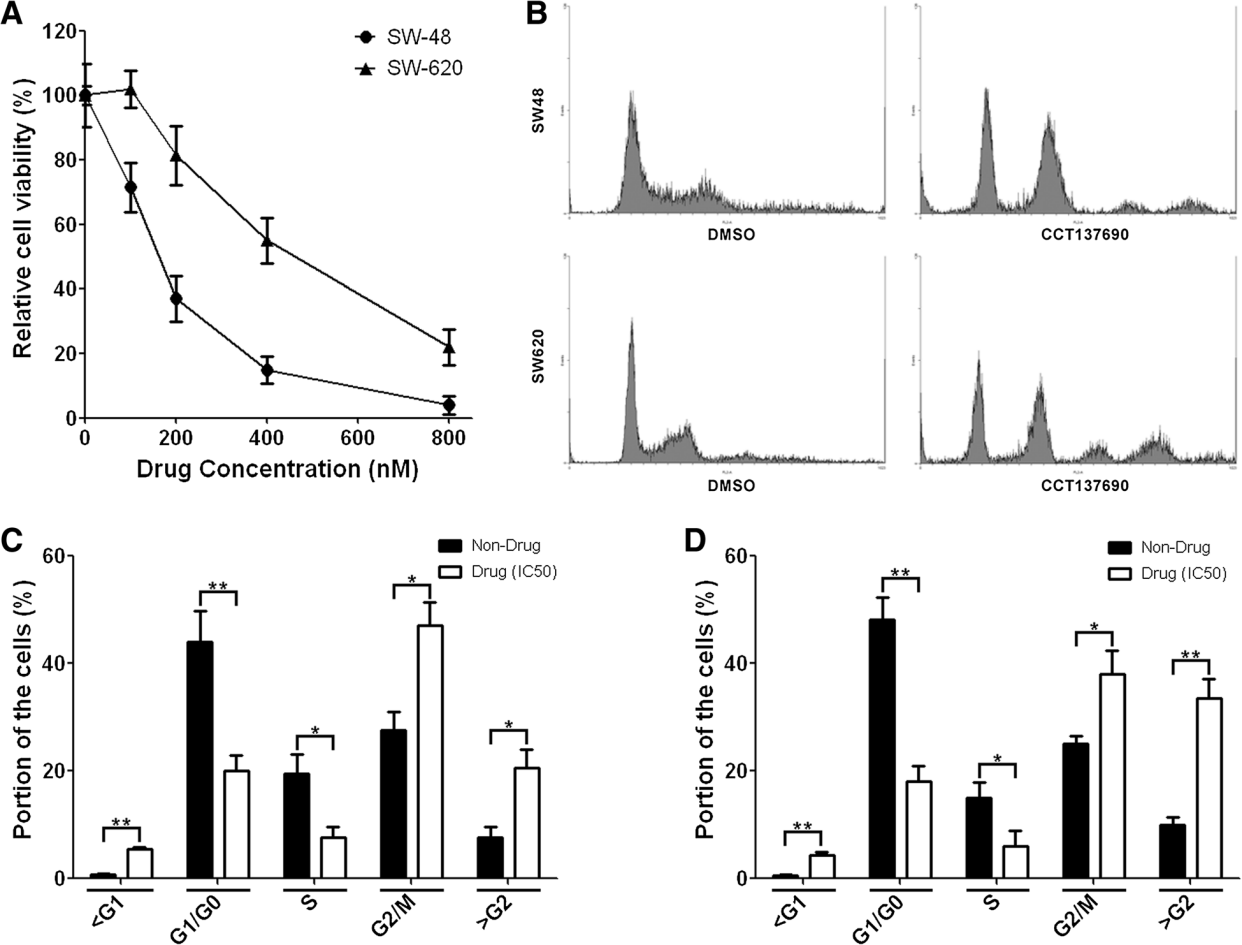
**The effects of CCT37690 on proliferation of SW48 and SW620 cells. A**, relative cell viabilities of SW48 and SW620 cells treated by different concentrations of CCT137690 were measured by using MTT assay. **B**, representative figures of cell cycle assays; SW48 with (157 nM) or without drug-treatment, SW620 with (430 nM) or without drug-treatment. **C** and **D**, quantification of cell cycle assays; **C** for SW48, **D** for SW620, mean ± SD, (n = 3).

**Figure 2:**
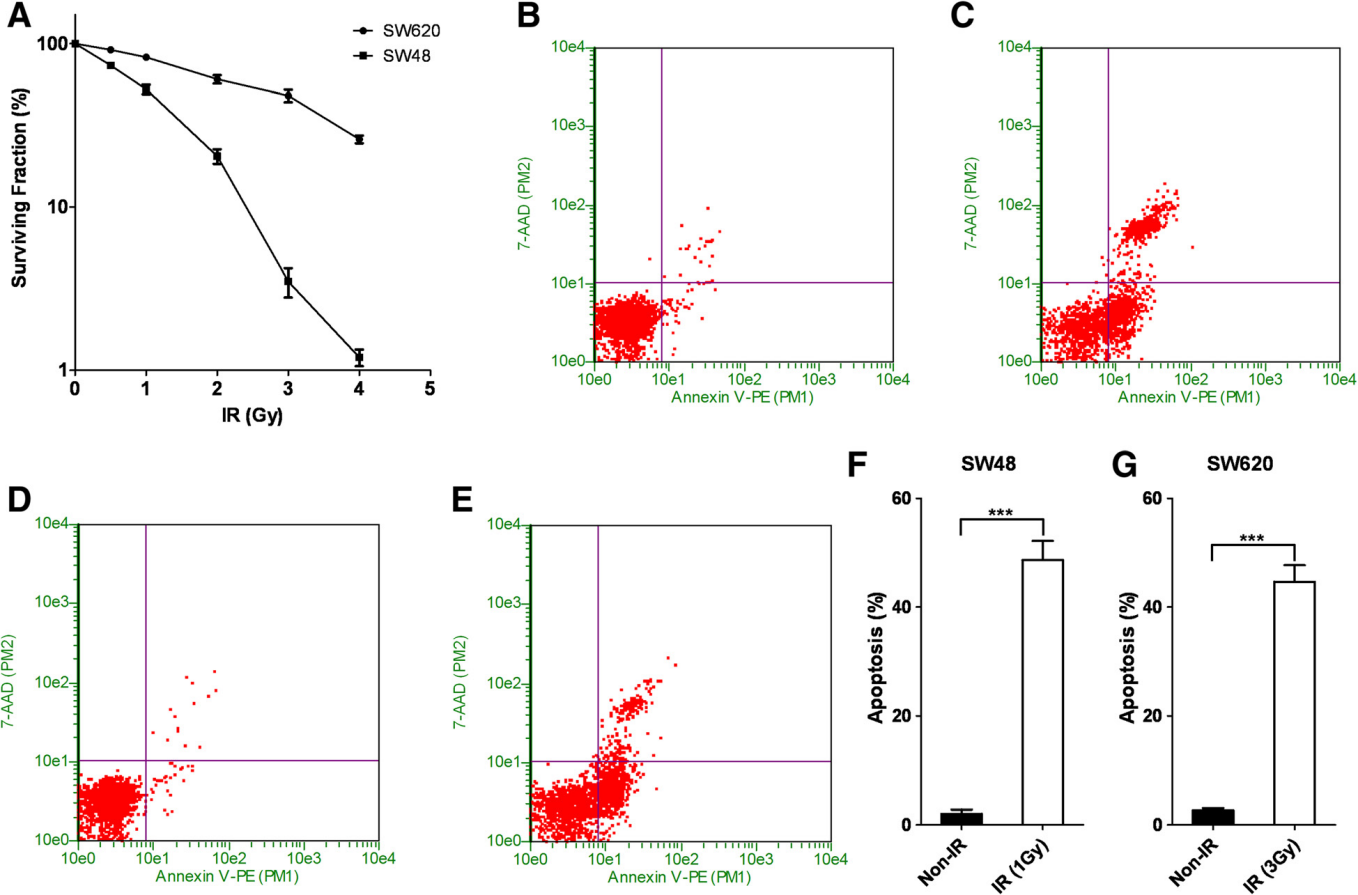
**The effects of radiation on SW48 and SW620 cells. A**, surviving curves of cells after ionization-radiation. **B** and **C**, representative figures of Guava Nexin assay of SW48 cells, **B** is for cells without radiation while **C** is for cells with 1 Gy of radiation. **D** and **E**, representative figures of Guava Nexin assay of SW620 cells, **D** is for cells without radiation while **E** is for cells with 3 Gy of radiation. **F** and **G**, quantification of Guava Nexin assays, **F** for SW48, **G** for SW620, mean ± SD, (n = 3).

**Figure 3:**
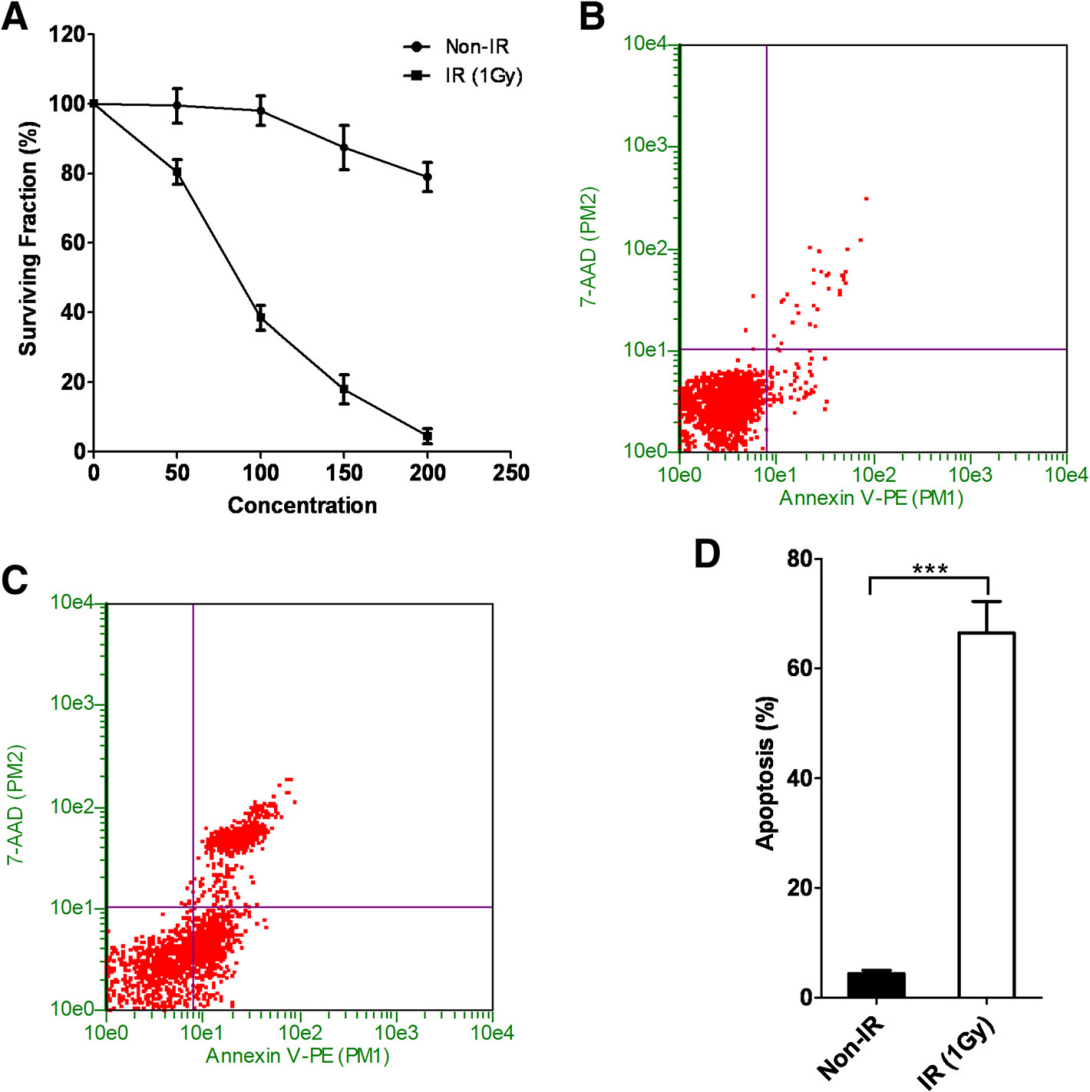
**Low-dose radiation can increase the sensitivity of SW620 to CCT137690. A**, SW620 cells were treated with different concentrations of CCT137690 for 48 hours before they were treated with (1 Gy) or without radiation. **B** and **C**, representative figures of Guava Nexin assays of cells pre-treated with 100 nM of CCT137690 and then treated with **(C)** or without **(B)** radiation. **D**, quantification of Guava Nexin assays on above cells, mean ± SD, (n = 3).

**Figure 4:**
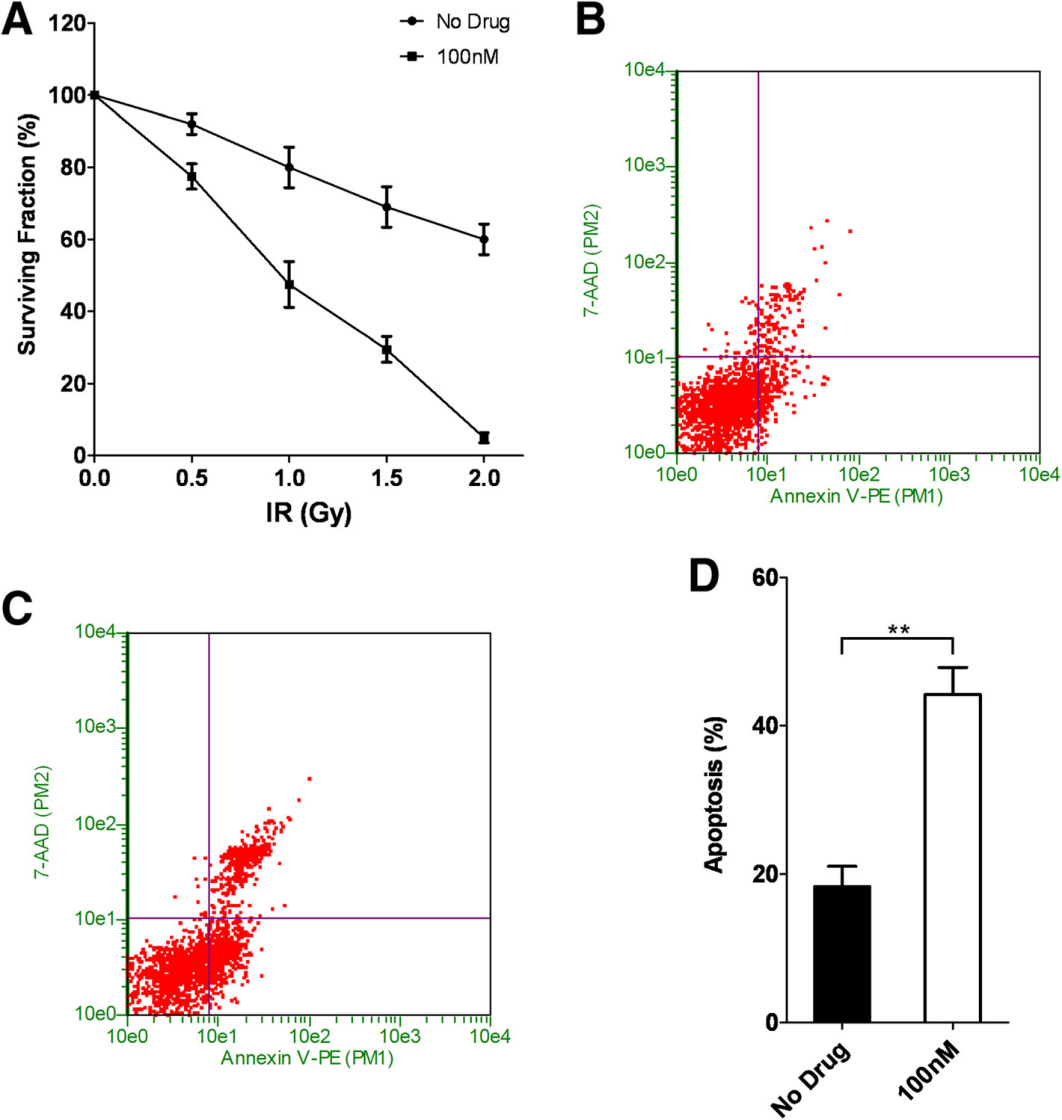
**Low dosage of CCT137690 sensitizes SW620 cells to radiation. A**, SW620 cells (with or without drug treatment (100 nM)) were exposed to different dosages of radiation. **B** and **C**, representative figures of Guava Nexin assays of cells (with **(C)** or without **(B)** drug treatment) exposed to 1 Gy of radiation. **D**, quantification of Guava Nexin assays on above cells, mean ± SD, (n = 3).

In the current study, we sought to identify whether the combination of radiotherapy and inhibition of Aurora kinases could exert a synergistic inhibitory effect on colorectal cancer cell growth. To test this hypothesis, we first characterized the sensitivity of two different colorectal cancer cell lines SW-48 and SW-620 to an Aurora kinase inhibitor, CCT137690. We show that both SW-48 and SW-620 exhibit dose-dependent responses to CCT137690 treatment. Moreover, we found that SW-620 is relatively more resistant to CCT137690 treatment as compared to SW-48 cells as manifested by a higher IC50 (430 nM vs 157 nM) (Figure 1A). In addition, when cells were treated with CCT137690 at their respective IC50, we observed cell cycle perturbations in both cell lines. There was a lower proportion of cells in G_1_/G_0_ and S phase, and a higher proportion of cells in G_2_/M and > G2 (Figure 1B-D).To determine sensitivity of the cell lines to radiotherapy, GUAVA assay was employed and revealed that radiation was able to induce significant apoptosis in both SW-48 and SW-620 cell lines (Figure 2B-G). However, the cell lines displayed different sensitivities to IR: SW-620 cells exhibits a higher resistance to radiation compared to SW-48 cells (Figure 2A). Indeed, higher amounts of radiation (3 Gy vs 1 Gy) were required for a similar apoptosis response in SW-620 cell vs SW-48 cell (Figure 2F and G).To test whether there is any synergistic effects of radiotherapy and Aurora kinase inhibition, SW620 cells were treated with different concentrations of CCT137690 before they were treated with a low-dose radiation (1 Gy) or without IR (Figure 3A). Our data suggested that a low-dose radiation dramatically enhances the inhibitory effect of CCT137690 on cell growth. 100 nM of CCT137690 has very limited effects on SW620. But surprisingly, when combined with radiation, a big proportion of the cells treated with CCT137690 died through apoptosis (Figure 3D).In light of these observations, we ascertained whether low-dose CCT137690 pretreatment could exert a similar effect to radiation. As shown in Figure 4A, 100 nM of CCT137690 pre-treatment dramatically decreases survival of SW620 cells exposed to radiation. In line with this notion, we also found that CCT137690 pre-treatment dramatically enhances radiation-induced apoptosis (Figure 4B-D).

**Figure 5:**
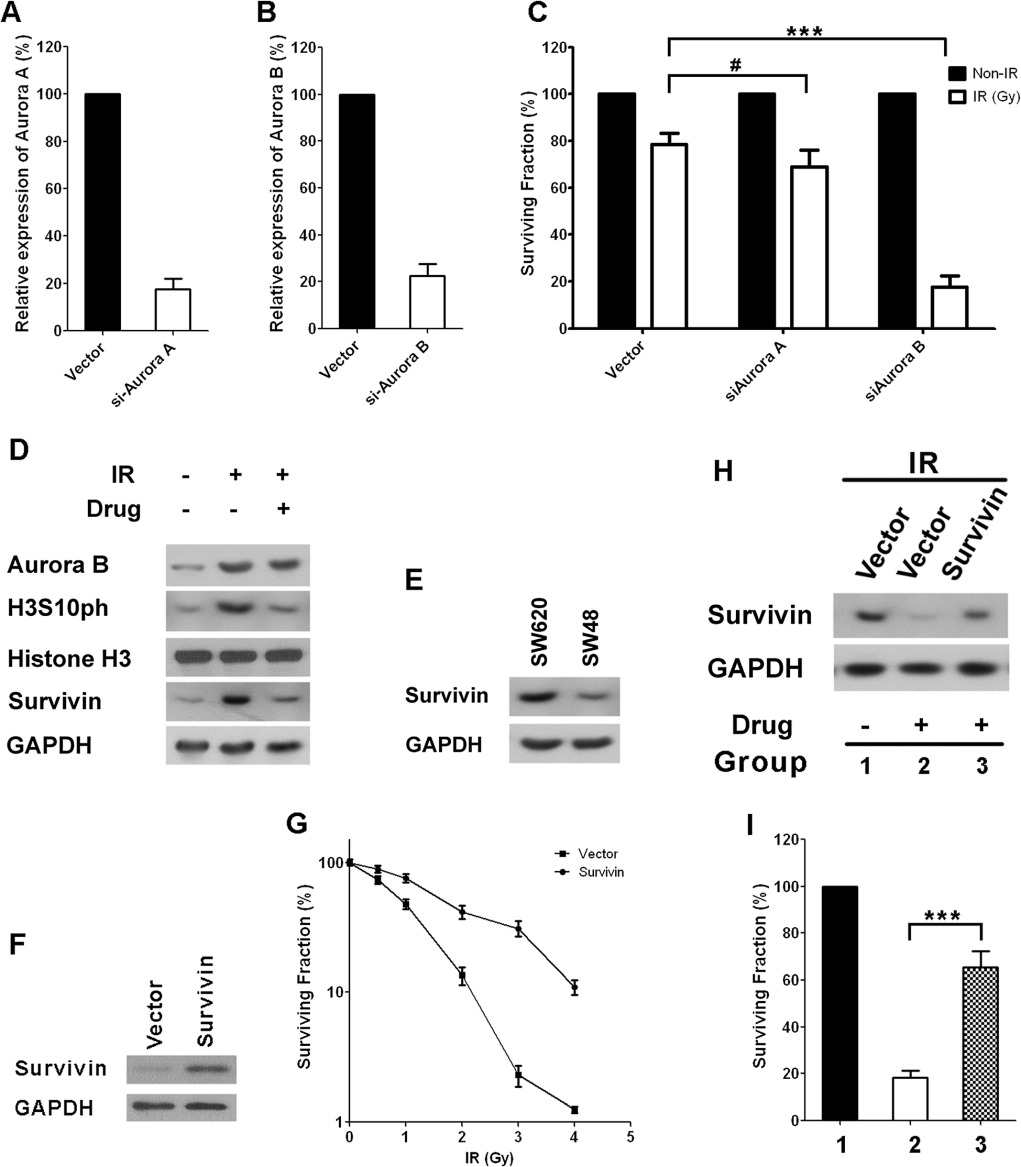
**The mechanism underlying the synergic action between CCT137690 and radiation. A** and **B**, relative expression of the indicated genes in SW620 cells treated by control or siRNA of the indicated target, mean ± SD, (n = 3). **C**, surviving fractions of siRNA transfected cells (from **A**, **B**) with or without radiation. **D**, Western blot analysis of Aurora B, Ser10-phosphorated Histone H3, Histone H3, survivin and GAPDH in cells with indicated treatments (IR means 1 Gy, drug = 100 nM). **E**, the protein levels of survivin and GAPDH in SW48 and SW620 cells. **F**, protein levels of survivin and GAPDH in SW48 cells with or without survivin over-expression. **G**, the surviving fractions of SW48 cells with or without survivin over-expression upon radiation. **H**, the protein levels of survivin and GAPDH in cells with or without survivin over-expression under indicated treatments (IR =1 Gy, drug = 100 nM). **I**, the surviving fractions of cells described in **H**, mean ± SD, (n = 3).

Since CCT137690 inhibits the activities of both Aurora A and Aurora B, we wished to clarify whether the synergistic effects of CCT137690 to radiation were due to inhibition of Aurora A or Aurora B. We therefore used siRNA to deplete either Aurora A or Aurora B in SW620 cells (Figure 5A and B). As shown in Figure 5C, only knockdown of Aurora B dramatically decreases cell survival following radiation (p < 0.001) while knockdown of Aurora A does not exert a similar effect (Figure 5C). We found that radiation induced Aurora B protein expression and correspondingly higher Aurora B activity, as manifested by increased phosphorylation of histone H3 (at serine 10) (Figure 5D). In addition, survivin is a reported target of Aurora B-mediated phosphorylation, and it inhibits caspase activation thereby mediating cell survival through inhibiting apoptosis [34]. We corroborated these results by showing that radiation induced higher Aurora B activity and correspondingly increased survivin protein expression. However, when cells were additionally treated with CCT137690 to inhibit activity of Aurora B, the protein levels of survivin decreased (Figure 5D). Since survivin is a very important anti-apoptotic protein, the decrease of survivin may explain the synergistic effects between radiation and CCT137690. Consistent with this notion, survivin protein expression in SW-48 cells was much lower than that in SW-620 cells (Figure 5E), which may explain the different sensitivities of these cells to radiation. To confirm this point, we managed to over-express survivin in SW48 cells (Figure 5F). As expected, survivin over-expression significantly increases the surviving rates of the cells after radiation (Figure 5G). To further confirm the central role of Aurora B-survivn signaling pathway in regulating survival upon radiation, we treated SW620 cells with CCT137690 before radiation, lower survivin protein level correlates with lower surviving rate after radiation (Figure 5H and I). In addition, survivin over-expression in drug-treated cells greatly ameliorates radiation induced cell death (Figure 5H and I) further confirmed our hypothesis.

## Discussion

Radiotherapy stands a major adjunctive therapeutic option for colorectal cancer management. Although there have been intensive investigations on the optimal regimen of radiotherapy for this lethal disease, very limited success have been made during the past several decades.

CRC is notorious for being refractory to both chemotherapy and radiotherapy. Thus investigators are particularly interested in characterizing novel molecule targets which exert regulatory effects on sensitivity to radiochemotherapy in CRC patients. Positive results from these studies might be clinically important since untoward side effects from radiotherapy or chemotherapy stands as major concerns for clinicians in tumor management and sensitizers of radiochemotherapy may help to reduce dosage load and associated toxic side effects.

In light of this notion, we started to search for potential sensitization targets for radiotherapy of CRC subjects and we found that there is a recent growing interest in the role of Aurora B and cancer biology.

In terms of synergistic effect of Aurora B inhibition and radiotherapy sensitivity, a previous study has shown that Aurora B inactivation sensitizes mesothelioma cells [27]. In addition, Aurora B inhibition also potently suppresses repopulation during fractionated irradiation of human lung cancer cells [28].In the current study, we first show that SW-620 colorectal cancer cells are relatively resistant to Aurora B inhibition by CCT137690 and to radiation (Figures 1 and 2). However, we found that the combination of Aurora B inhibition and radiation exerts synergistic effects on cancer cell growth inhibition. Our results showed that low-dose radiation (1 Gy) greatly exaggerates the growth inhibitory effect of CCT137690 on SW-620 cells (Figure 3A), as well as a low-dose of CCT137690 dramatically increases the sensitivity of cells to radiation (Figure 4A).

Our observations provide a good proof of concept that both chemotherapy and radiotherapy doses could be greatly lowered by taking the advantages of synergistic effects of those two interventions. This could be translated into the clinic where the expectation is that there would be less adverse side-effects and better patient tolerance at lower doses. These findings are especially important given that CT137690 has a narrow safety margin.

In terms of understanding of the mechanism by which inhibiting Aurora B increases radiosensitivity of CRC cells, we found that Aurora B-survivin pathway may be involved (Figure 5). These findings are consistent with several reports showing the association of Aurora B and survivin in context of CRC. For example, Tuncel et al. reported that nuclear Aurora B and cytoplasmic survivin expression is involved in lymph node metastasis of colorectal cancer [16]. Moreover, Aurora-survivin signaling machinery has been implicated in other cancers such as myelodysplasia [35], chronic lymphocytic leukemia [36], head and neck squamous cell cancer [37]. In this regard, we observed that forced-expression of survivin dramatically ameliorates Aurora B-inhibition induced CRC cell death in the context of radiation (Figure 5I).

Taken together, our results for the first time showed that Aurora B inhibition, via CCT137690, and radiation exposure may play synergistic effects in colorectal cancer death. Taking advantage of this synergistic effect, a lower dose of radiation exposure and/or chemical exposure is required for cancer cell death induction, which may have significant clinical implications for CRC management.

## Competing interests

All authors agreed that there is nothing to disclose for the current study.

## Authors’ contributions

XYW and WTL designed the current study, performed the experiments, collected the data and drafted the manuscript. QHC, CC, ZWC, ZX, WSL provided scientific inputs for study design and technical support for GUAVA assay, construct cloning and radiation treatment. FKL and XQY mentored the whole project, drafted and revised the manuscript. All authors read and approved the final manuscript.
